# In-shoe plantar temperature, normal and shear stress relationships during gait and rest periods for people living with and without diabetes

**DOI:** 10.1038/s41598-025-91934-9

**Published:** 2025-03-14

**Authors:** Athia Haron, Lutong Li, Jiawei Shuang, Chaofan Lin, Maedeh Mansoubi, Xiyu Shi, Daniel Horn, Neil Reeves, Frank Bowling, Katherine Bradbury, Andrew Eccles, Safak Dogan, Helen Dawes, Glen Cooper, Andrew Weightman

**Affiliations:** 1 School of Engineering, University of Manchester, Manchester, UK; 2 College of Mechanical Engineering, Yangzhou University, Yangzhou, 225127 People’s Republic of China; 3 Medical School, NIHR Exeter BRC, University of Exeter, Exeter, UK; 4 Institute for Digital Technologies, Loughborough University London, Queen Elizabeth Olympic Park, Here East, London, UK; 5 Survey of Health, Ageing, and Retirement in Europe (SHARE Berlin Institute), Berlin, Germany; 6 Lancaster Medical School, Faculty of Health and Medicine, Lancaster University, Lancaster, UK; 7 Manchester University NHS Foundation Trust within the Departments of Diabetes and Vascular Surgery, Manchester, UK; 8 School of Psychology, University of Southampton, Southampton, UK; 9 School of Social Work & Social Policy, University of Strathclyde, Glasgow, UK

**Keywords:** Diabetes, Diabetes complications, Mechanical engineering, Translational research

## Abstract

Diabetic foot ulcers (DFUs) are a common complication of diabetes. This study aims to investigate the relationships between in-shoe plantar temperature, normal and shear stress during walking and rest periods for participants with and without diabetes. For this purpose, a novel temperature, normal and shear stress sensing system was developed and embedded in an insole at the hallux, first metatarsal head and calcaneus region. Ten participants living with diabetes with no history of previous ulceration and ten healthy participants were recruited. Participants walked on a treadmill for 15 min and then rested for 20 min wearing the sensing insole. Results showed high correlation (*Spearman’s r*_*s*_ ≥ 0.917) between heat energy, total plantar temperature change, during walking and strain energy, cumulative stress squared in all participants. Importantly, between-group comparisons showed indications of thermal regulation differences in participants with and without diabetes, with the first metatarsal head site showing significantly higher temperature at the end of the active period (*P* = 0.0097) although walking speed and mechanical stress were similar. This research demonstrates for the first time the correlation between strain energy and heat energy in-shoe during gait. Further research is needed to quantify relationships and investigate thermal regulation as a mechanism for DFU formation.

**Supplementary Information:**

The online version contains supplementary material available at 10.1038/s41598-025-91934-9.

## Introduction

Diabetes presents a global healthcare challenge, with diabetic foot ulcers (DFUs) as a severe complication^1^. It is also highly prevalent, with up to 34% of people living with diabetes potentially developing DFUs in their lifetime, leading to high recurrence rates, a diminished independence and quality of life, and substantial costs on both the individual and healthcare systems^1,2^. By implementing preventive measures such as pre-DFU formation detection strategies, the incidence of DFUs can be minimized, and treatment optimised leading to better outcomes for people living with diabetes and healthcare systems alike.

Walking is essential for personal independence and maintaining physical and mental health, and personal independence. For people living with diabetes, ulcers may form on areas of the foot that experience pressure while walking^2^, and develop at a prevalence of between 38–57%^3^. Ledoux et al. showed that the regions of the foot with most ulcers formation were at the hallux (40% of ulcers formed), the metatarsal heads (26%), and the heel (21%), and were sites that coincide with the highest peak plantar pressure^4^. To address this paradoxical problem many research studies have used in-shoe normal stress sensors (including commercially available sensing systems like Tekscan’s *F-scan*, Novel GmbH’s *Pedar*, and XSensors’ *X4*^5^) to identify areas of high plantar pressure with a view to managing the risk of DFU formation. Until recently there was very limited research into in-shoe shear measurement, due to lack of sensing technology, with no commercial system available. Although the first in-shoe shear system developed for research was introduced in 1992^6^, the second documented system was in 2013^7^, 21 years after the first system. This area has seen increased focus over the last 8 years (2016–2024) with only four (to the authors knowledge) sensing systems developed^8–11^. However, these healthcare technologies have not translated into the clinical care pathway due to low accuracy, partial stress field measurement and a general lack of understanding of the aetiology of DFU.

**Fig. 1:**
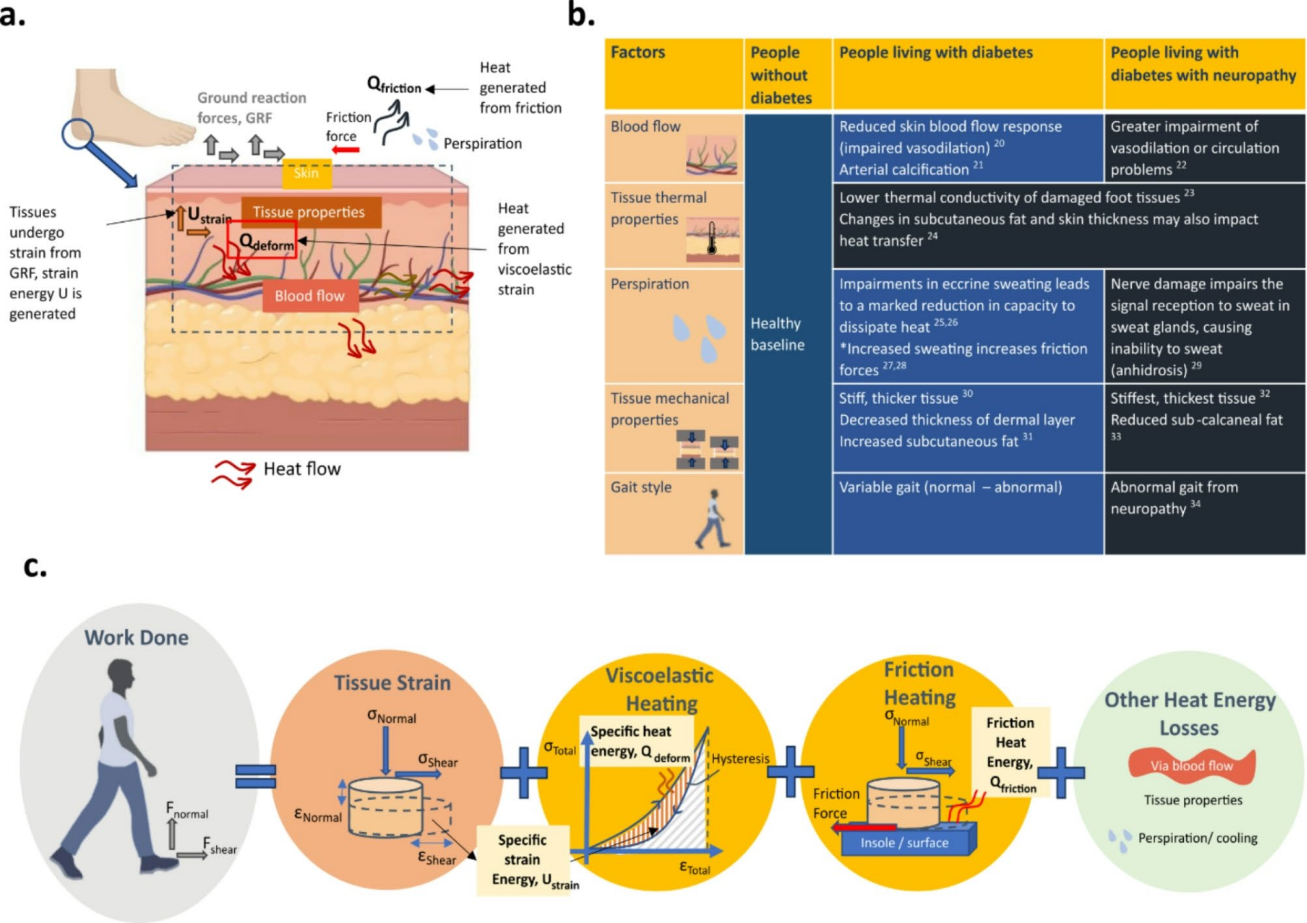
Illustration of the conservation of energy at the foot during gait activity. (**a**) Energy exchange illustration at the skin due to mechanical loading, illustrating the pathways of energy transfer at the skin level. (**b**) Factors affecting heat transfer and thermoregulation and their differences in people living with diabetes, with neuropathy and without diabetes)^23–37^. (**c**) Illustrative equation of the energy exchange at the foot due to mechanical loading (or gait).

Research studies have focused on plantar temperature and its relation to DFU risk detection through its link to inflammation and potential ulcer development in static conditions^12–14^. An increase in local dermal temperature precedes DFU^12,15^, with a 2.2 °C temperature difference between corresponding sites indicating imminent ulceration risk^16,17^. Barefoot studies on gait activity and plantar temperatures have shown variations in temperature rise time in different healthy age groups^18^ and temperature differences in people living with diabetes with and without Charcot foot^19^. Our previous studies of healthy individuals^20^ and of people living with diabetes^18^ that solely measured plantar temperature indicate that plantar temperature is linked to walking activity showing differences in individuals living with and without diabetes. Therefore, it is possible that the mechanisms that lead to changes in temperature could play a significant role in DFU prediction, prevention, and management. While early studies have explored these links through in vitro experiments^21^, there is no quantifiable link between mechanical loading and temperature change, especially in the diabetic foot. A single study has explored the effect of gait speed on plantar temperature, but this was on young healthy participants and only qualitatively described a proportional relationship^22^. Studies with participants living with diabetes that explore the links between mechanical loading and temperature change have yet to be undertaken. The complex relationship of temperature changes in response to mechanical stress created through activities of daily living is poorly understood, and to what extent could it be a contributory factor to DFU development is not known. The complex mechanisms affecting plantar tissue temperature are likely linked to mechanical stress, inflammation, tissue damage, and vascularization (as illustrated in Fig. 1a and b)^23–37^. People living with diabetes are likely to have an altered temperature response in each of these mechanisms when resting, during and after activity, potentially leading to abnormal plantar tissue changes^33^. An altered response to rest, activity and recovery may lead to tissue damage especially in individuals where there is a history of ulceration, neuropathy, or poor vascularisation.

Recent investigations into the full mechanical stress state during gait have focused on measuring normal and shear stresses of the foot in people living with and without diabetes, but have not included simultaneous temperature measurements^8–10,29,38,39^. Lack of concurrent measurement hinders further exploration of the relationship between temperature and the full mechanical stress state of the foot and longer term understanding of the mechanobiology of DFU formation. One study found no correlations between peak pressures, peak shear stress, and post-walk temperature measurements^40^. However, this important finding measured temperatures before and after gait, therefore did not study relationships between the dynamic changes during gait. Reddy et al. measured dynamic changes of temperature during gait, but didn’t explore the relationships behind these mechanisms^20^. Additionally, the work reported in the literature does not explore links between temperature change and mechanical cyclic loading which is exhibited in viscoelastic materials such as those found in human plantar tissue^41^.

Walking or other activities of daily living create ground reaction forces which act on the plantar tissue. This interaction could be considered in terms of energy, that the work done by the ground reaction forces transfers to energy stored within the plantar tissue in terms of strain energy and heat energy. Plantar tissue is a bio-composite multilayered structure with complex mechanics but it is known to have viscoelastic properties^41^. Viscoelastic materials have hysteresis in their stress-strain characteristic from loading and unloading as strain energy is converted into heat energy^21^. This energy exchange is not within a closed system as some of the heat energy will leave the local plantar tissue and be lost to either the surroundings or transferred to other body tissues via conduction or through blood circulation, a factor often impaired in people with diabetes. This energy exchange is shown in Fig. 1 and described by Eq. (1).

1 $$\:W=\:{U}_{strain}+\:{Q}_{deform}+\:{Q}_{friction}+\:{E}_{loss}$$

where W is the input energy or work done on the plantar tissue, $\:{U}_{strain}$ is the strain energy stored in the plantar tissue, $\:{Q}_{deform}$ is the heat energy stored in the plantar tissue caused by the viscoelastic heating, $\:{Q}_{friction}$ is the heat energy generated in the tissue from friction and $\:{E}_{loss}$ is the energy lost to the surroundings. All quantities have the units of joules.

Previous research has looked at the peak temperature of plantar tissue and peak plantar stress and found no association, however, Eq. (1) suggests there may be relationships between strain energy and heat energy (detailed mathematical explanation within the methods section).

The aim of this study was to investigate the relationships between in-shoe plantar temperature, normal and shear stress during walking and rest periods for people living with and without diabetes.

From previous research and what we know regarding how viscoelastic materials behave under cyclic loading, we hypothesise the following:

### Hypothesis 1

There will be no association between peak plantar temperatures and peak plantar stresses, as we cannot directly compare stress and temperature as indicated through dimensional analysis; instead, we must consider energy relationships.

### Hypothesis 2

There will be a correlation between strain energy and heat energy in the plantar tissue. The first law of thermodynamics regarding the conservation of energy (in this instance Eq. 7) would imply a change in plantar temperature and plantar total stress squared will correlate in individuals.

### Hypothesis 3

Participants living with diabetes’ plantar temperatures will exhibit higher increases and cool at a slower rate than participants without diabetes.

## Methods

### Study design

Reporting is aligned to the Strengthening the Reporting of Observational studies in Epidemiology (STROBE) reporting guidelines for observational studies and checklist^42^.

This study was repeated measures (during walking and rest periods) mixed within- and between-subjects experimental design.

### Ethics and participant recruitment

The study received approval from the NHS Health Research Authority and Health and Care Research Wales (HCRW) Ethics Committee (REC reference: 22/NW/0216), and all participants provided written informed consent prior to participation. Trial Registration number: NCT05865353. All methods were performed in accordance with the relevant guidelines and regulations.

The sample size (*N* = 10) was selected to meet proof-of-concept study requirements, providing preliminary data on intervention feasibility and efficacy prior to larger-scale investigations^43^. Two groups of participants were recruited, 10 living with diabetes (herein referred to as *group living with diabetes*, D) and 10 without diabetes (herein referred to as *control group*, C).

Participants were included in the study if they met the inclusion criteria and did not meet any of the exclusion criteria of the study (see Supplementary Table 1 for the full list of inclusion and exclusion criteria). Data from eight participants in the group living with diabetes and nine participants in the control group were analysed, as participants who could not complete the trials, or where there were problems with data collection, were discarded from the study. The demographics of the participants that completed the trial in this study are shown in Table 1. There were no differences in the main demographics of the participants completing and not completing the trial.

**Table 1:** Summary of demographics for the participants included in the study.

Group	Gender	Number	Average BMI	BMI category*	Average age (years)	Average height (m)	Average weight (kg)
Control (C)	Male	4	25.7 ± 2.8	Overweight	49.8 ± 10.2	1.79 ± 0.03	82.1 ± 6.4
	Female	5	27.4 ± 7.8	Overweight	56.0 ± 20.2	1.69 ± 0.10	77.4 ± 16.6
	Combined	9	26.6 ± 5.8	Overweight	53.2 ± 15.9	1.74 ± 0.10	79.5 ± 12.6
Living with diabetes (D)	Male	7	29.6 ± 4.3	Overweight	54.6 ± 17.3	1.76 ± 0.10	90.5 ± 11.8
	Female	1	31.3 ± 0	Obese	67.0 ± 0.0	1.62 ± 0.00	82.2 ± 0.0
	Combined	8	29.6 ± 4.0	Overweight	56.1 ± 16.6	1.74 ± 0.10	89.4 ± 11.3

Values shown are mean and standard deviation (mean ± standard deviation).

*BMI Categories: Healthy 18.5 ≤ BMI ≤ 24.9; Overweight 25 ≤ BMI ≤ 29.9; Obese BMI ≥ 30; BMI category is based on the NHS guidelines for most adults (www.nhs.uk/conditions/obesity).

The consenting participants were invited to attend two sessions: (Gait Lab Visit 1) baseline assessment and the (Gait Lab Visit 2) gait data collection.

### Gait lab visit 1: baseline assessment

Participants first attended a baseline assessment where after completing demographic data collection, anthropometric data (weight, height, age, and foot sizes) was collected, and anatomical landmark locations were determined using manual palpation of the foot. A skin safe marker pen was used to mark the anatomical landmarks (of the first metatarsal head, hallux and calcaneus bones) on the sole of the foot. This was conducted by a single researcher to maintain consistency of measurement. Participants then stepped onto white paper to transfer the marked positions, and the outline of the foot was also marked on the paper. The anatomical landmark positions and foot outline were used to position the sensors and determine the insole size respectively (personalised sensing insoles). The personalised sensing insoles were manufactured before the gait data collection in the second visit. Participants in the group living with diabetes had their feet assessed for presence of loss of sensation via a 10 g monofilament test prior to the walking activities. Their fitness to perform the activities were assessed by asking the participants to conduct a two-minute treadmill walk while wearing a pair of mock sensor insoles (insoles without any embedded sensors) in prophylactic shoes (Sponarind 97308, Finn Comfort Inc. Hassfurt, Bavaria, Germany). The prophylactic shoes had leather inner and outer material, removable insole, a flat profile mid sole and outer sole, a wide toe-box, with Velcro fasteners to ensure an optimal fit (with the same person to fit the shoes in the participants each time) and to allow individual adjustment of the width. Their ability to complete the walk and general comfort were assessed (via observation and verbal check of comfort) after the short gait activity.

### Temperature, normal and shear (TNS) sensing insoles

A custom TNS sensor was designed consisting of three sensors measuring temperature, normal stress and shear stress. The TNS insole system utilised the novel tri-axial (normal, medial-lateral [ML] and anterior-posterior [AP] shear) stress sensor fully described in our previous study^11^. But briefly, the TNS sensors consisted of a commercial temperature sensor (JT Ultra-Thin Film NTC Thermistor, ATC Semitec Ltd, Cheshire, United Kingdom, ± 0.15 °C accuracy) which was placed above a normal (commercial normal stress sensor, Flexiforce A301, Tekscan, Norwood, Massachusetts) and shear stress sensor (3-element strain Rosette gauge arrangement, 1-RY81-3/120, Hottinger Bruel & Kjaer UK Ltd, Royston, England), with errors of up to ± 18.0 kPa for the Flexiforce sensor^44^, and up to ± 1.8 kPa error for the shear stress sensor^11^ for the respective measurement ranges, giving a combined average error of up to ± 10.3 kPa. The combined accuracy for the normal and shear sensor measurement range is thus between 96 and 99% (or error of 1–4%)^11^. Our errors are comparable to commercial in-shoe plantar normal stress sensing systems, which show root mean square errors between RMSE 2.6 kPa–27.0 kPa^45^. Only measurements more than twice the accuracy of the sensors (of temperature and stress sensors) were considered a measurable value that reflects the actual recorded data rather than values that may be from measurement error.

**Fig. 2:**
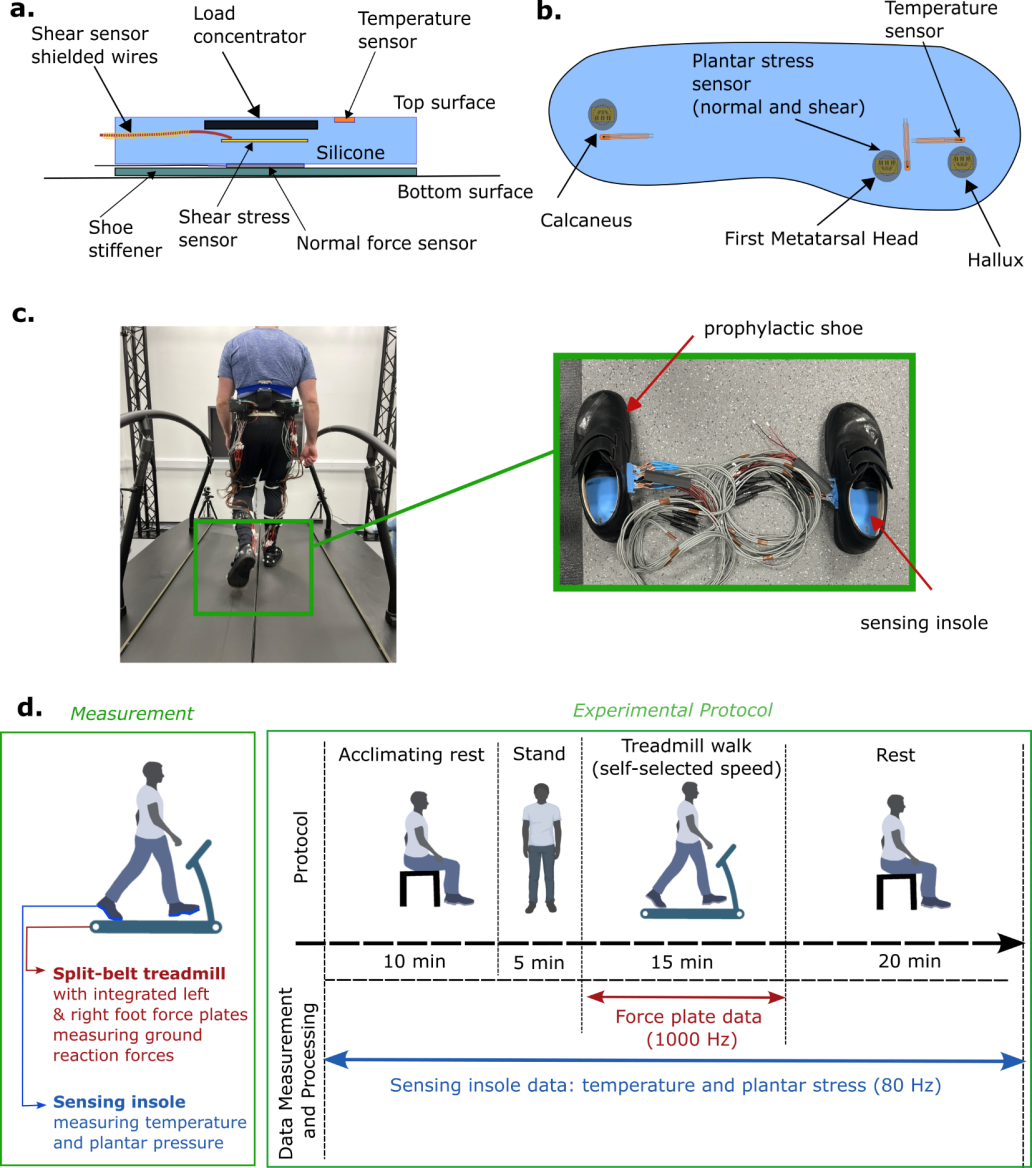
Experimental setup, protocol and sensing insole system. (**a**) Cross section of the TNS sensor showing the two-dimensional locations (width and depth) of the individual sensors (temperature, normal stress and shear stress sensors) relative to each other (temperature sensor accurate up to 0.15 °C; normal and shear stress sensor combined error up to ± 10.3 kPa and > 97% reliability, see Haron et al., 2024). (**b**) Fully assembled sensing insole with three TNS sensors placed at three key sensing locations (calcaneus, first metatarsal head and hallux). (**c**) Experimental setup, where a participant (left image) wore the sensing insoles with the prophylactic shoes (right image) and walked on the instrumented split belt treadmill at their chosen self-selected pace. (**d**) Experiment protocol and data collection for the study.

The TNS sensor are shown in Fig. 2a. Three TNS sensors (co-located normal and shear sensors, described in Haron et al. (2024), with commercial temperature sensors) were embedded in a 10 mm thick silicone insole at anatomical locations based on identified anatomical bony landmarks from the baseline visit (first metatarsal head, hallux, and calcaneus, Fig. 2b). The temperature, normal and shear sensing insole (TNS insole) data was collected at 80 Hz and were collated via a bespoke microcontroller (Teensy 4.1, PJRC, Portland, Oregon, USA) data acquisition system whilst wirelessly monitored on a computer.

The calibration of the insoles for the individual participants followed the procedures fully described in Haron et al., as this was found to be important for reliable and accurate measurement^11^.

All data collected by the TNS insole was then parsed and pre-processed to obtain calibrated, final measurement results via custom scripts in MATLAB (v2023, The Mathworks Inc., Natick Massachusetts, USA). The plantar stress data was minimally pre-processed before finalizing into calibrated stress measurements according to Haron et al. (2024). This pre-processing stage removed noise spikes from the signal, if it was present, which was less than 0.05% of the total data collected. This was outworked using the *filloutlier* function with the ‘quartile’ outlier detection option to remove ‘quartile’ outliers which were elements more than 1.5 interquartile ranges above the upper quartile (75th percentile) or below the lower quartile (25th percentile) and correcting DC offsets. Data from each foot were analysed separately.

Plantar temperature data was smoothed using a moving median smoothing function over 100 samples (or 1.25 s) to aid in removing noisy signals from the foot losing contact with the insole surface where the temperature was measured.

The variables of stress (normal and shear) measurements from the TNS insole used for further investigation were: (i) median-average peak stress (kPa), (ii) Pressure Time Integral, PTI (kPa·s) and the (iii) cumulative sum of stress squared M(Pa)^2^. The temperature variables investigated in this study were: (i) maximum temperatures at the end of walking, (ii) change in temperature between start to end of 15-minute walking, and (iii) change in temperature between start to end of 20-minute resting.

### Gait lab visit 2: gait data collection protocol and setup

Participants wore the sensing insoles in the specialist prophylactic shoes along with thermally conductive socks (Silversock, Carnation Footcare, Oldbury, United Kingdom) and given sports tights (Run tights, Karrimor Ltd., England, UK) and shorts (Sondico Core shorts, Lovell Sports Ltd., England, UK) to wear for the entire experimental protocol. Ambient room temperature was measured at the beginning of the experimental protocol for each participant, and the average for all visits was 21.4° ± 1.3° (mean ± standard deviation).

The participants then sat down wearing the prophylactic shoes and personalised sensing insoles for 10 min at the beginning of the test (acclimating rest) followed by 5 min of standing. They were then asked to walk on a split-belt treadmill (M-Gait, Motek Medical BV, Amsterdam, Netherlands, Fig. 2c) at their self-selected speed for 15 min, with a 20-minute rest period after walking (see Fig. 2d for the full protocol). Participants were asked to walk at their self-selected speed to provide the most natural gait representation^46^, a common metric used by other researches in this field (for example, in the study by Hosein and Lord, 2000). The treadmill collected normal force data from each foot at 1000 Hz via integrated force plates.

### Treadmill force plate data

Cadence (steps per minute), walking speed (ms^− 1^) and ground reaction forces (N) were collected from the force plates on the treadmill. Self-selected walking speeds were determined from the treadmill measurements and assumed to be equal to the horizontal velocity of the foot centre of mass at mid-stance. The value provided was the mean from all left and all right foot stance phases within the two minutes of the baseline assessment. Cadence was calculated from the number of steps (determined from peak ground reaction force for each foot) within one minute.

### Data analysis

All measured data analyses were carried out in MATLAB unless explicitly stated otherwise. Peak temperature occurred and was measured at the end of walking period. Peak stresses were the median of all peak stresses of five minutes in the middle of the walking period.

The equations for the data analysed within the study are as follows:

From Eq. (1), if we assume that most of the heat energy created in the plantar tissue is likely to come from viscoelastic heating, it would be logical that strain energy, $\:{U}_{strain}$, will be related to heat energy, $\:{Q}_{deform}$, from the theory of conservation of energy, as strain is associated with a temperature rise in the material. Also, we assume that the plantar tissue could be represented by a Kelvin-Voigt model, then its strain-strain characteristic would be given by Eq. (2).

2 $$\:\sigma\:=E\cdot\:\epsilon\:+\eta\:\cdot\:\dot{\epsilon\:}$$

where $\sigma$ (Pa) is the stress in the material, $\:E$ (Pa) is the stiffness of the elastic part of the material, η (Nsm^–2^) is the viscosity of the material and $\:\epsilon\:$ (unitless) and $\:\dot{\epsilon\:}$ (s^− 1^) are the strain and strain rate of the material, respectively.

The strain energy of the material, $\:{U}_{strain}$, is given by Eq. (3).

3 $$\:{U}_{strain}=V\underset{0}{\overset{{\epsilon\:}_{Max}}{\int\:}}\sigma\:\cdot\:d\epsilon\:=V\underset{0}{\overset{{\epsilon\:}_{Max}}{\int\:}}\left[E\epsilon\:+\eta\:\dot{\epsilon\:}\right]d\epsilon\:=V\left[\frac{1}{2}E{\epsilon\:}^{2}+\eta\:\frac{{\epsilon\:}^{2}}{2t}\right]$$

where $\:V$ (m^3^) is the volume of the material. If we substitute this volume in for the strain component for the elastic and viscous part of the material, Eq. (3) can be written in terms of stress as Eq. (4)

4 $$\:{U}_{strain}=V\left[\frac{{\sigma\:}^{2}}{2E}+\frac{{\sigma\:}^{2}t}{2\eta\:}\right]$$

where *t* (s) is the time taken to deform the material.

Heat energy in the material from the deformation, $\:{Q}_{deform}$, is given by Eq. (5).

5 $$\:{Q}_{deform}=m\cdot\:c\cdot\:\varDelta\:T=V\cdot\:\rho\:\cdot\:c\cdot\:\varDelta\:T$$

where $\:m$ (kg) is the mass of the material, $\:c$ (Jkg^− 1^ °C^− 1^) is the specific heat capacity of the material, $\:\varDelta\:T$ (K) is the temperature rise in the material and $\:\rho\:$ (kgm^− 3^) is the density of the material.

Assuming that deformation strain energy converts into heat energy, we could equate Eqs. (4 and 5) to produce Eq. (6).

6 $$\:V\cdot\:{\sigma\:}^{2}\left[\frac{1}{2E}+\frac{t}{2\eta\:}\right]=V\left[\rho\:\cdot\:c\cdot\:\varDelta\:T\right]$$

Equation (6) gives a simplified relationship between stress and temperature as the real situation involves multiple layers of tissue and other mechanisms, some of which are illustrated in Fig. 1c. Although multi-faceted, Eq. 6 shows that change in temperature is proportional to stress squared shown in Eq. (7).

7 $$\:\varDelta\:T\propto\:{\sigma\:}^{2}(a+b\cdot\:t)$$

where $\:a$ and $\:b$ are constants.

From Eq. (7), heat energy is related to strain energy by correlations between change in temperature and total stress squared.

Total strain energy can be determined using the Maximum Shear Strain Energy Theory (*Hencky – Von Mises Theory*^47,48^). Strain energy is composed of two forms: volume changes caused by normal strains, $\:{U}_{volume}$, and distortion caused by shear strain, $\:{U}_{distortion}$.

The total strain energy ($\:{U}_{total\:strain}$) is described as

8 $$\:{\:U}_{total\:strain}=\:{U}_{distortion}+\:{U}_{volume}$$

The cumulative sum of the values of stress squared can be written as the discrete sum of stress squared evaluated at each time point *t* ($\:{\sigma\:}_{t}^{2}$), of the measurement from start ($\:t=\:{t}_{0}$) to end ($\:t=\:{t}_{f}$) of measurement. The results are then summed and multiplied with the time between consecutive measurements ($\:\varDelta\:t$) to give a cumulative total ($\:S$):

9 $$\:S=\:\sum\:_{t=\:{t}_{0}}^{{t}_{f}}{\sigma\:}_{t}^{2}\varDelta\:t$$

The strain energy equation (Eq. (4)) can be written as proportionate to the cumulative sum of the values of the stress components squared, and thus the total strain energy (Eq. (8)), equation becomes:

10 $$\:{\:S}_{total\:stress}\propto\:\:{S}_{shear}+\:{S}_{normal}$$

Therefore, the study investigated the correlation between heat energy (as the change in temperature from start to end of walking) and strain energy (as the cumulative sum of stress squared) in three forms:

(i) Change in temperature correlation with strain energy from distortion (shear stress) $\:\varDelta\:T\propto\:$ $\:{S}_{shear}$
(ii) Change in temperature correlation with strain energy from volume changes (normal stress) $\:\varDelta\:T\propto\:$ $\:{S}_{normal}$
(iii) Change in temperature correlation with the total strain energy $\:{\:\varDelta\:T\propto\:S}_{total\:stress}$.

A summary table of key terminology can be found in supplementary material (Supplementary Table 2).

### Statistical analysis

All data were coded anonymously to ensure participant confidentiality. The Shapiro-Wilk test was used to determine normality of the dataset, in which it confirmed most of the dataset were not normally distributed, as expected for studies with small sample sizes. For this reason, statistical correlation analyses to estimate the strength of associations used non-parametric tests. Linear regression was utilised to estimate the strength of relationships. Statistical analyses were conducted using the MATLAB statistical toolbox, with results expressed (where appropriate) as median and interquartile ranges, p-values and a median with a 95% confidence interval (CI).

A linear regression model for the participants similar to Yavuz et al.^3^ was made to estimate the extent of the linear relationship found between peak temperatures and peak stress in either group (people living with and without diabetes) separately.

A Spearman’s rank correlation analysis was made between change in temperature and cumulative sum of stresses squared for all individuals in the study. Each foot was treated separately, as to accommodate minor differences in foot and gait pathologies within individuals (an *intra-participant energy analysis*).

Mann-Whitney U tests were conducted to compare medians of the temperature changes or cumulative sum of stresses squared between participants living with diabetes and the control participants without. A Linear regression model was made to determine whether a linear relationship between change in temperature (proportional to heat energy) and cumulative sum of stresses squared (proportional to strain energy) can be found in either group separately. This was also used to determine whether there are differences in the regressions between participants living with diabetes and the control participants.

## Results

### General measurement summary (gait, temperature and stress measurement)

Median and interquartile ranges [median (IQR)] for walking speed for the group living with diabetes (D) and control group (C) were 0.80 (0.25) ms^− 1^ and 0.72 (0.44) ms^− 1^ respectively. Cadences for the two groups (D and C) were 95 (15) steps per minute and 87 (23) steps per minute respectively (Supplementary Table 3). No statistically significant differences were found in these parameters between the two groups (p-values of *P* = 0.59 for walking speed, and *P* = 0.24 for cadence, Mann Whitney U).

Stress and temperature were continuously monitored throughout gait and the rest period. Results from the stress and temperature measurements, as well as the percentage difference of the measurements between the group living with diabetes and the control group are shown in Table 2. The data collected was used to test the three hypotheses.

Average (median) peak stresses, measured as the median of all peaks for five minutes in the middle of the walking period, were not significantly different between the participant groups (*P* > 0.05). However, the medians indicated that the average peak stresses were generally lower in the group living with diabetes (D) than the control group (C). The average peak stresses were up to 26% lower (Table 2, first metatarsal head, medial shear stress), except for two instances: calcaneus peak normal stress (6% higher) and hallux lateral shear stress (9% higher). Pressure time integrals (PTI) and the cumulative sum of stresses did not always follow the peak stress percentage difference trends (AP shear PTI at 4 and 6% higher in participants living with diabetes at the hallux and first metatarsal head respectively, for example), but match in trends to each other (cumulative sum of AP shear stress squared at 9% and 10% higher at the hallux and first metatarsal head in the participants living with diabetes).

Pressure time integral (PTI) trends between the groups indicated a difference of foot loading proportions between the groups, with a larger difference between normal stress PTI of the calcaneus and first metatarsal head of 744 kPa·s in the control group, compared to a difference of 286 kPa·s in the group living with diabetes (Table 2).

Participants in the group living with diabetes (D) had higher median peak temperatures at the end of the walking period compared to the control group (C) at all measured anatomical locations, with a significant difference at the first metatarsal head of 1.0 °C (*P* = 0.0286, Mann Whitney U, Cohens D effect size > 0.8).

Two sub groups were identified in the study: (i) two participants in the group living with diabetes who reported more than 50% loss of sensation from the 10 g monofilament test at either foot, and (ii) four participants in the control group (two males and two females), who had BMIs within the healthy range (< 24.9 and > 18.5). All participants had an increase in temperature at the end of the 15-minute walking period, of between + 2.0 °C to + 4.9 °C (Table 2, Supplementary Fig. 1a). For both groups, the lowest temperature increase was seen at the Hallux, and the largest temperature increase at the calcaneus. During the rest period that followed, both groups showed either a significant slowing of increase in temperature, or complete reduction in temperature (or cooling), Supplementary Fig. 1b.

No significant differences were found in the median and peak stresses between the group living with diabetes and control group (or between the group of participants living with diabetes who reported more than 50% of loss of sensation at the foot, and the control group participants with healthy BMI.

**Table 2:** Summary of the median values (average) of peak temperatures, peak stresses, temperature change, cumulative sum of stress squared during walking and temperature change during rest for the two groups (participants living with diabetes, D; control participants without diabetes, C).

	Group	Living with diabetes (D)			Control (C)			Percentage differences $\:\left(\frac{\varvec{D}-\varvec{C}}{\varvec{C}}\times\:100\varvec{\%}\right)$		
	Location	Hallux	First Metatarsal Head	Calcaneus	Hallux	First Metatarsal Head	Calcaneus	Hallux	First Metatarsal Head	Calcaneus
Normal stress	Average peak normal stress (kPa)	177.6 (117.4)	147.7 (93.9)	286.6 (125.5)	231.6 (96.8)	175.4 (111.7)	269.7 (71.7)	-23	-16	6
	Normal Stress Pressure Time Integral (kPa·s)	591.2 (585.5)	700.1 (839.7)	985.7 (771.1)	908.6 (1153.7)	723.4 (489.3)	1467.7 (585.7)	-35	-3	-33
	Cumulative Sum of Normal stress squared M(Pa)^2^	279.4 (772.0)	489.5 (944.1)	1152.3 (1106.0)	800.8 (1537.1)	634.5 (857.3)	1510.4 (922.4)	-65	-23	-24
Anterior-posterior [AP] shear stress	Average peak posterior shear stress (kPa)	63.6 (43.8)	57.7 (51.5)	47.8 (96.6)	81.2 (67.2)	73.2 (48.4)	96.9 (125)	-22	-21	-51
	Average peak anterior shear stress (kPa)	55.2 (76.8)	48.9 (20)	49.1 (41.6)	57.1 (87.4)	54.1 (33.6)	81.3 (113.8)	-3	-10	-40
	AP Pressure Time Integral (kPa·s)	367.2 (386.0)	425.0 (222.2)	330.1 (536.3)	359.7 (414.5)	408.0 (286.3)	619.7 (917)	2	4	-47
	Cumulative Sum of AP shear stress squared M(Pa)^2^	91.1 (201.8)	118.3 (92.5)	65.0 (238.8)	83.9 (215.0)	107.5 (124.6)	210.6 (750.1)	9	10	-69
Medial-lateral [ML] shear stress	Average peak lateral shear stress (kPa)	34.4 (20.9)	23.9 (17.6)	27.5 (17.6)	31.6 (74.2)	32.2 (37.4)	31.5 (41.1)	9	-26	-13
	Average peak medial shear stress (kPa)	27.8 (18.4)	20.4 (14.9)	26 (31.5)	38.7 (36.4)	28.3 (16.6)	48.3 (78.3)	-28	-28	-46
	ML Pressure Time Integral (kPa·s)	237.3 (163.7)	162.2 (150.9)	183.9 (138.9)	234.6 (491.4)	208.8 (194.8)	239.6 (607.2)	1	-22	-23
	Cumulative Sum of ML shear stress squared M(Pa)^2^	32.8 (52.6)	15.9 (22.9)	21.1 (39.0)	27.1 (179.0)	22.9 (43.1)	34.8 (301.6)	21	-31	-39
Total stress (combined normal and shear stress)	Cumulative Sum of total stress squared M(Pa)^2^	881.4 (757.5)	758.8 (993.4)	1388.5 (1413.5)	1362.1 (2508.4)	763.8 (672.3)	2389.7 (2080.8)	-35	-1	-42
Temperature	Maximum temperature at the end of walking (°C)	30.2 (2.3)	29.6 (3.8)	30.4 (3.5)	29.9 (2.3)	28.6 (0.8)	30 (1.7)	1	3	1
	Temperature Change from start to end of walking (°C)	2.4 (1.2)	2.8 (1.3)	3.6 (1.9)	2.0 (1.6)	2.1 (0.6)	4.9 (3.2)	100	-67	-150
	Temperature Change from start to end of rest (°C)	0.6 (0.9)	0.2 (0.9)	0.1 (0.6)	0.3 (0.8)	0.6 (1.0)	-0.2 (1.3)	20	33	-27

Percentage difference between the two groups is shown in the final three columns on the right side of the table.

Stress measurement combined error up to ± 10.3 kPa; Temperature values accurate up to ± 0.15 °C.

Positive percentages = participants with diabetes higher than control; Negative percentages = participants with diabetes lower than control.

### Is peak stress related to peak temperature?

No significant relationship could be found between peak stresses and peak temperature, with weak linear correlations observed (Pearson’s R^2^ = 0.0001–0.128, *P* > 0.05, Supplementary Table 4) between the peak stresses (for all stresses: normal, AP shear, and ML shear) and peak temperatures during walking at all three of the measured anatomical locations of the foot.

### Correlation between cumulative sum of stress squared (proportional to strain energy) and temperature change (proportional to heat energy generated)

Spearman’s rank analysis showed significant and strong correlation values (Spearman’s correlation coefficient *r*_*s*_ ≥ 0.917, *P* < 0.05; Table 3) between the cumulative sum of stress squared and the temperature change (example data in Supplementary Fig. 2). This was observed in all participants, at all three of the measured anatomical locations of each foot, and for every type of plantar stress: normal, AP shear, ML shear and total.

**Table 3:** Intra participant energy analysis correlation results of the cumulative sum of stresses squared with temperature change for every participant at the three measured anatomical locations of the foot.

Individual correlation		Cumulative sum of normal stress squared with change in temperature			Cumulative sum of AP shear stress squared with change in temperature			Cumulative sum of ML shear stress squared with change in temperature			Cumulative sum of total stress squared with change in temperature			
Participant (foot)		MH	HA	HE	MH	HA	HE	MH	HA	HE	MH	HA	HE	
Group living with diabetes (*N* = 8, *n* = 16)	1(L)	0.999	1.000	1.000	0.999	1.000	1.000	0.999	1.000	1.000	0.999	1.000	1.000	All p values < 0.05, meaning all correlations were statistically significant
	1(R)	1.000	0.993	0.978	1.000	0.993	0.978	1.000	0.993	0.978	1.000	0.993	0.978	
	2(L)	0.975	0.950	0.975	0.975	0.917	0.975	0.975	0.917	0.975	0.975	0.917	0.975	
	4(L)	1.000	0.999	0.999	1.000	0.999	0.999	1.000	0.999	0.999	1.000	0.999	0.999	
	4(R)	1.000	0.998	0.999	1.000	0.998	0.999	1.000	0.998	0.999	1.000	0.998	0.999	
	5(L)	0.992	0.993	0.959	1.000	0.993	0.978	1.000	0.993	0.978	1.000	0.993	0.978	
	5(R)	0.998	1.000	1.000	1.000	1.000	1.000	1.000	1.000	1.000	1.000	1.000	1.000	
	6(R)	1.000	0.998	0.999	1.000	0.998	0.999	1.000	0.998	0.999	1.000	0.998	0.999	
	7(L)	1.000	0.994	1.000	1.000	0.994	1.000	1.000	0.994	1.000	1.000	0.994	1.000	
	7(R)	1.000	0.947	0.998	1.000	0.947	0.998	1.000	0.947	0.998	1.000	0.947	0.998	
	8(L)	0.981	0.994	0.992	0.981	0.994	0.992	0.981	0.994	0.992	0.981	0.994	0.992	
	8(R)	0.996	0.994	0.948	0.996	0.994	0.948	0.996	0.994	0.948	0.996	0.994	0.948	
	9(L)	1.000	0.999	0.997	1.000	0.999	0.997	1.000	0.999	0.997	1.000	0.999	0.997	
	9(R)	1.000	0.923	0.994	1.000	0.923	0.994	1.000	0.923	0.994	1.000	0.923	0.994	
Control group participants (*N* = 9, *n* = 18)	1(L)	0.997	1.000	0.998	0.997	1.000	0.998	0.997	1.000	0.998	0.997	1.000	0.998	
	1(R)	0.999	0.999	0.999	0.999	0.999	0.999	0.999	0.999	0.999	0.999	0.999	0.999	
	2(L)	1.000	0.989	0.993	1.000	0.989	0.993	1.000	0.989	0.993	1.000	0.989	0.993	
	2(R)	1.000	0.995	0.997	1.000	0.995	0.997	1.000	0.995	0.997	1.000	0.995	0.997	
	3(L)	1.000	1.000	1.000	1.000	1.000	1.000	1.000	1.000	1.000	1.000	1.000	1.000	
	3(R)	1.000	0.994	1.000	1.000	0.994	1.000	1.000	0.994	1.000	1.000	0.994	1.000	
	4(L)	1.000	0.991	1.000	1.000	0.991	1.000	1.000	0.991	1.000	1.000	0.991	1.000	
	4(R)	1.000	1.000	1.000	1.000	1.000	1.000	1.000	1.000	1.000	1.000	1.000	1.000	
	6(L)	0.997	1.000	1.000	0.997	1.000	1.000	0.997	1.000	1.000	0.997	1.000	1.000	
	6(R)	1.000	1.000	1.000	1.000	1.000	1.000	1.000	1.000	1.000	1.000	1.000	1.000	
	7(L)	1.000	0.992	0.999	1.000	0.992	0.999	1.000	0.992	0.999	1.000	0.992	0.999	
	7(R)	0.999	0.998	0.999	0.999	0.998	0.999	0.999	0.998	0.999	0.999	0.998	0.999	
	8(L)	1.000	1.000	0.992	1.000	1.000	0.992	1.000	1.000	0.992	1.000	1.000	0.992	
	8(R)	1.000	0.998	0.996	1.000	0.998	0.996	1.000	0.998	0.996	1.000	0.998	0.996	
	9(L)	1.000	0.998	1.000	1.000	0.998	1.000	1.000	0.998	1.000	1.000	0.998	1.000	
	9(R)	1.000	0.999	1.000	1.000	0.999	1.000	1.000	0.999	1.000	1.000	0.999	1.000	
	10(L)	1.000	1.000	0.999	1.000	1.000	0.999	1.000	1.000	0.999	1.000	1.000	0.999	
	10(R)	1.000	1.000	0.999	1.000	1.000	0.999	1.000	1.000	0.999	1.000	1.000	0.999	

Results show high correlation values of r_s_ ≥ 0.917, and was significant *P* < 0.05 for all correlations.

MH: First metatarsal head; HA: Hallux; HE: Calcaneus (or heel).

N = number of participants; n = number of feet (left and right foot).

### Plantar temperature differences between participants living with diabetes and without diabetes during walking and resting

#### Larger temperature increase during gait in group living with diabetes

**Fig. 3:**
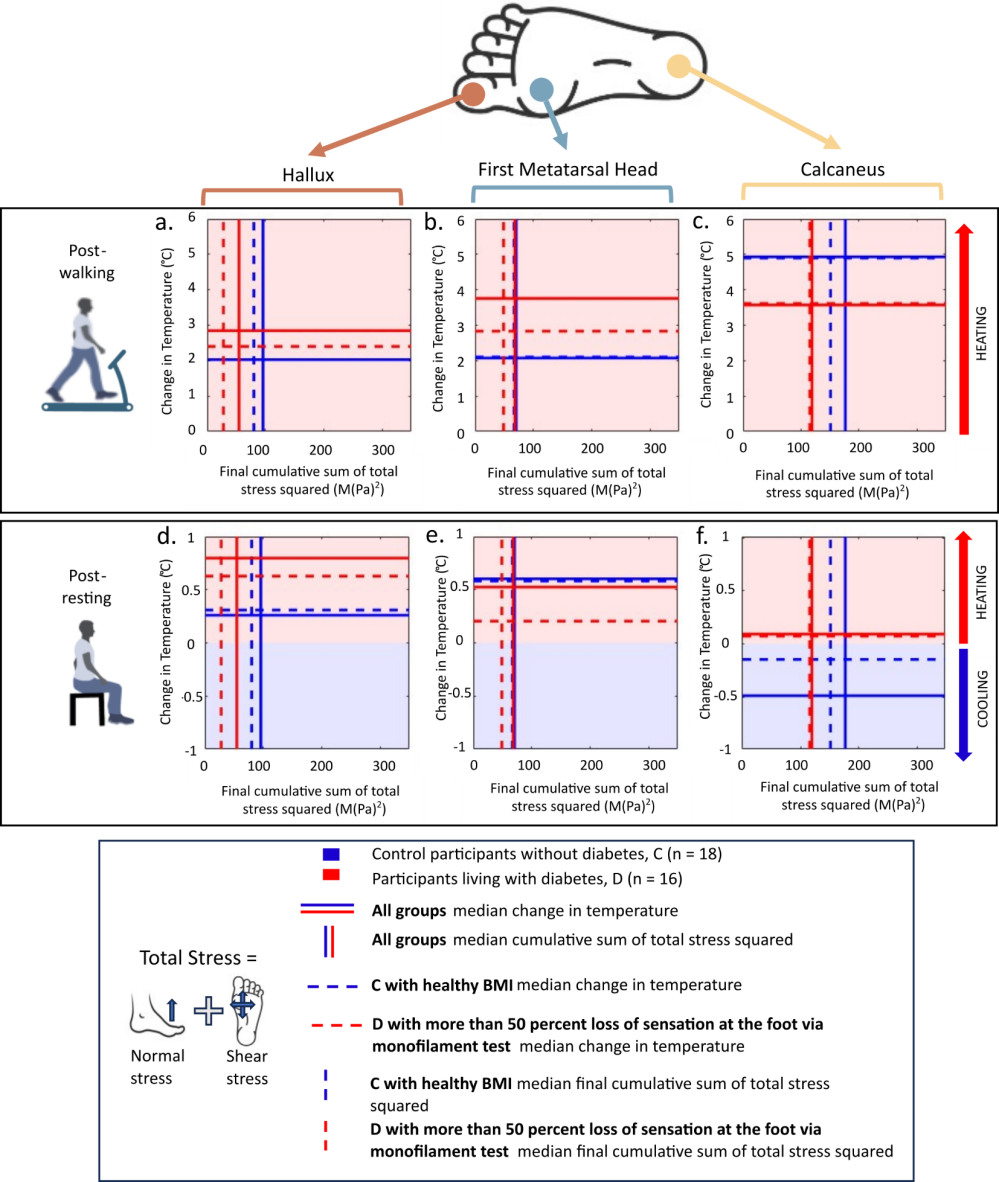
Medians of change in temperature (proportional to heat energy) and final cumulative sum of total stress squared (proportional to strain energy) between groups (living with diabetes and controls) for the three measured anatomical locations at the foot. Sub-groups are indicated as dashed lines, coloured regions show overall cooling/heating of the foot. (**a**–**c**) Show the medians of the post 15-minute walk, where all locations showed an increase in temperature (heating, up to 4.9 °C). (**d**–**f**) Show the medians of the post 20-minute rest, with a lower increase of change in temperature (first metatarsal head and hallux) or cooling (calcaneus, control group C). A key finding showed a significantly (*P*=0.0097, Mann Whitney U) higher temperature increase for the group living with diabetes despite lower or total cumulative stress squared compared to the control group (b).

Participants in the group living with diabetes (D) showed a larger median increase of temperature between the start and end of the walking period, than the control group without diabetes (C) at the hallux and first metatarsal head (Fig. 3), which were 18% and 35% higher, respectively (Table 4). The cumulative stresses for group living with diabetes (D) were lower at these locations compared to control group (C), 35% and 8% lower respectively. These differences were only statistically significant for the first metatarsal head (*p* = 0.0097, Mann-Whitney U, Cohen’s D effect size > 0.8).

Conversely, the temperature increase at the calcaneus was 1.3 °C higher in the control group (C) compared to the group living with diabetes (D). The differences in cumulative stress for group living with diabetes at the calcaneus compared to the control group was the lowest of the three sites. This cumulative stress in the group living with diabetes was 42% less than control group (*P* > 0.05, Mann-Whitney U, ~ 0.2 effect size of Cohen’s D).

The temperature differences were larger when comparing the sub-groups between participants that reported more than 50% loss of sensation from the monofilament tests and the participant in the control group with healthy BMI. A larger increase in temperature between start to end of walking was found in the group living with diabetes who reported more than 50% loss of sensation, which were 57% (at the first metatarsal head) and 40% (at the hallux) higher than the control group with healthy BMI. No statistical significance was found for these differences. These results are indicative as expected for the small sample size.

After the 20-minute rest period, the temperature changes at the participants feet continued to increase albeit at a slower rate than when walking (see Supplementary Fig. 1b). For all participants and at all the measured anatomical locations, there was a median increase in temperature of 0.6 °C from the start to end of the rest period as compared to the median increase of 2.5 °C in the walking period (Table 4). The only group where cooling was observed during rest was the control group at the calcaneus, decreasing by 0.5 °C. There was no cooling observed in group living with diabetes at any of the anatomical locations measured within the 20-min rest period.

**Table 4:**
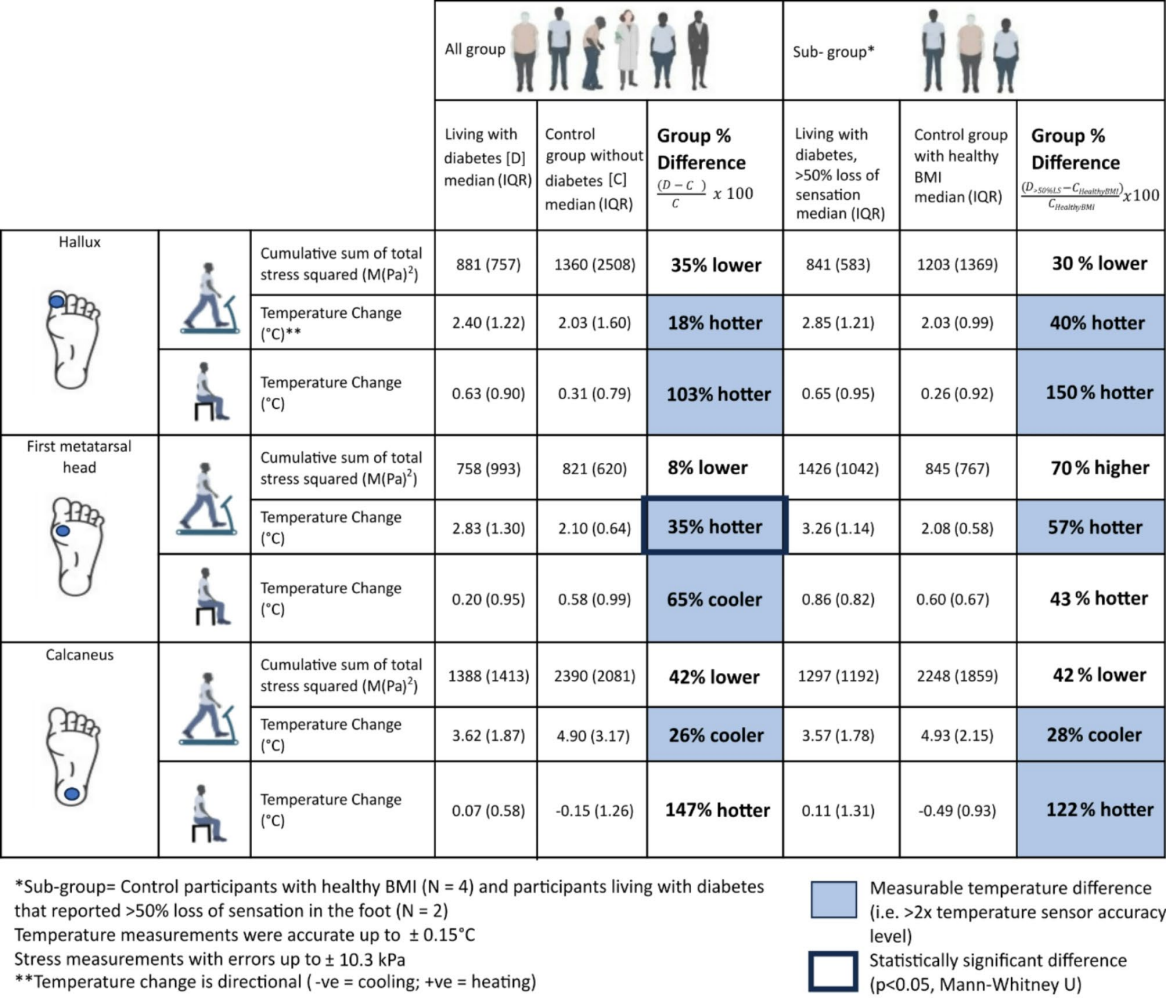
Summary of the median (and interquartile ranges IQR) values of cumulative sum of total stress squared (strain energy) and temperature change (proportional to heat energy) for both group of participants living with diabetes and the control group without diabetes.

Data is shown for the 15-minute walk and 20-min rest period. Data is divided into two larger groups of all (all participants) and sub groups (control participants with healthy BMI and participants with diabetes who reported more than 50% loss of sensation at the foot).

### Disruption of regression trends in people living with diabetes

A linear regression was also conducted between the total temperature change from the start to the end of walking (proportional to heat energy) and cumulative sum of total stress squared (proportional to strain energy). The results show, although not significantly, that the R^2^ values were higher (0.0001–0.3610, Supplementary Table 5) with this comparison compared to the peak analyses made in our first hypothesis (0.0001–0.1495, Supplementary Table 4).

**Fig. 4:**
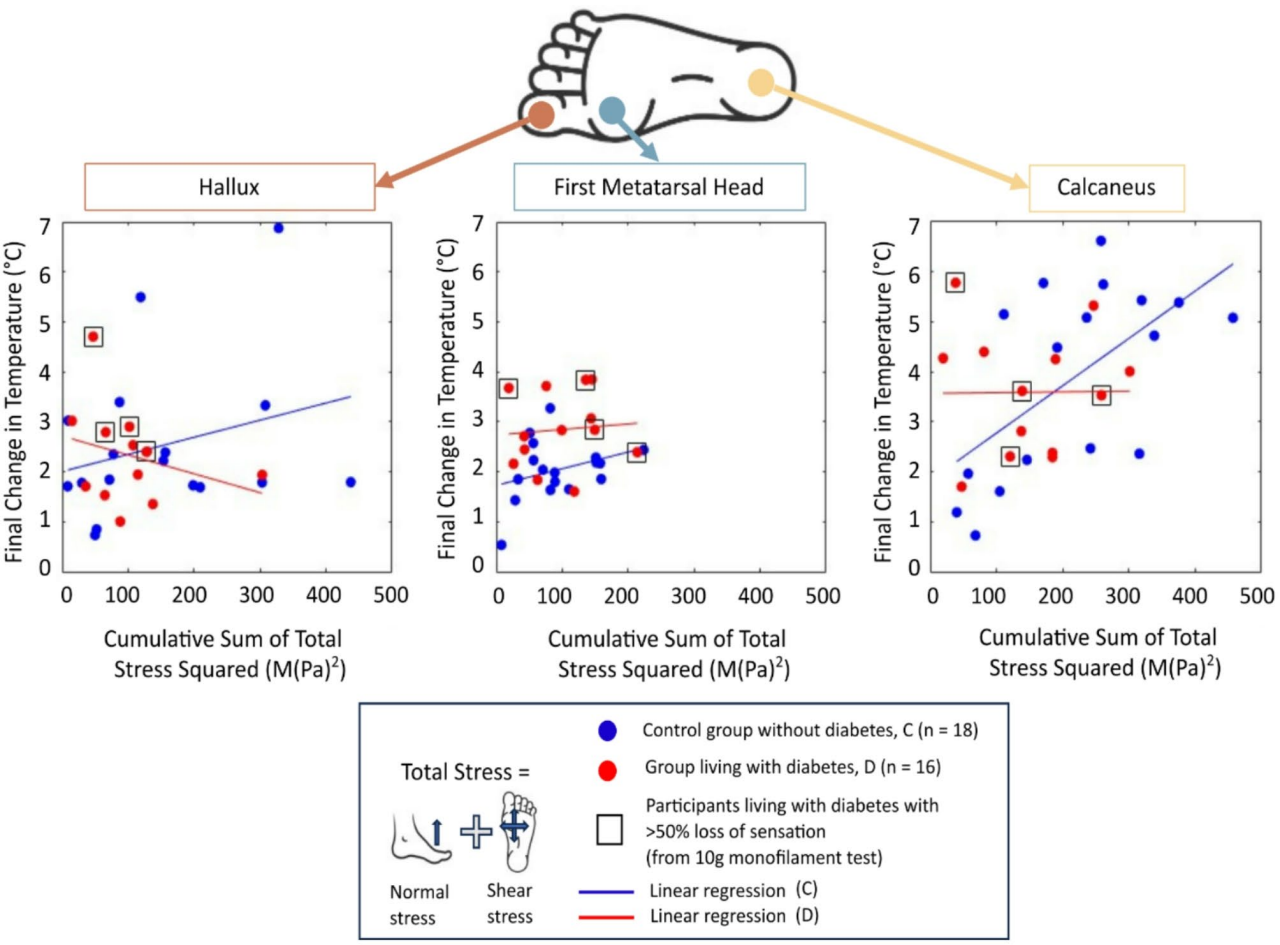
Linear regression lines between total cumulative stress squared (proportional to strain energy) and final change in temperature (proportional to heat energy) over the 15-minute treadmill walk. The two participants living with diabetes who had more than 50% loss of sensation determined by the 10 g monofilament test at the foot are indicated in the figure as black squares around the data point.

Furthermore, the R^2^ values were larger in the control group without diabetes (group C, R^2^ = 0.076–0.361, *P* > 0.05, Supplementary Table 4, Supplementary Table 5) compared to the group of participants living with diabetes (group D, R^2^ = 0.0001–0.081, *P* > 0.05, Supplementary Table 4, Supplementary Table 5). In the linear regression analysis between heat energy and strain energy especially, through visual inspection (Fig. 4), the slopes of the regression lines between group C and D intersect one another, indicating that the slopes are different. The regression lines showed positive slopes in the control group without diabetes for all the measured anatomical sites, compared to the group living with diabetes, where either a negative slope can be seen at the hallux, or low gradient slopes at the first metatarsal head and calcaneus.

For the regression lines made in the participants living with diabetes group, separating the participants that had more than 50% loss of sensation at the foot (sub group, shown as black squares in Fig. 3) showed that this group may have disrupted the linearity of the regression fit. However, the sample size for this sub group (*N* = 2) was not large enough to perform meaningful statistical analysis to support this assumption.

## Discussion

To the authors knowledge there have been no normal stress, shear stress and temperature sensing insoles developed and utilised for measurement of the relationship between these parameters for people living with and without diabetes outside of this work.

This study showed that an individual’s change in plantar temperature is highly correlated to the cumulative sum of all components of plantar stress squared from gait activity. As far as the authors are aware, this is the first study to investigate the relationships between in-shoe plantar stresses (normal, shear and total stress) and temperature during walking and to quantify their relationships in people living with and without diabetes.

The complex relationship between plantar temperature changes and mechanical stress from daily activities is not well understood in relation to diabetic foot ulcers (DFUs). We believe this interaction of temperature and stress could be a mechanism, in the formation of DFUs and warrants further investigation.

Generally, the results of the temperature measurements of the in-shoe TNS sensor matched previous research of temperature measurement during gait activity, in that plantar temperature increases with gait (Supplementary Fig. 1a), and then either levels off, or increases with residual heating or begins to decrease (Supplementary Fig. 1b) after a rest period^20,22^. This aligns with the principle of viscoelastic heating of plantar tissue shown in an in vitro porcine model^21^. The highest increase in plantar temperature post-gait for all participants was at the calcaneus (+ 4.9 °C) and lowest temperature increase at the foot extremities (hallux, metatarsal head) were also similar to a study of healthy subject studies walking at similar gait speed, where an increase in temperature post-gait of + 1.6 °C for the big toe and + 3.5 °C at the heel were observed^22^. Higher absolute peak temperatures were observed in the group living with diabetes in this research, which reflects the finding that plantar temperatures are generally higher than individuals without diabetes^49^.

There was a 1.0 °C significant difference (*P* = 0.0286) in absolute peak temperature post-gait at the first metatarsal head in the group living with diabetes compared to the control group without diabetes, which is a critical result, as higher temperatures may indicate tissue damage^12,15^ or impaired thermoregulation^26^.

Peak stresses in the group living with diabetes were lower than the control group. This is opposite to what is expected, or what has been observed in previous studies^50^, as the average weight of the group living with diabetes was higher than the control group. However, peak stress comparisons can suffer from outliers of isolated peak stress events. Peak stresses at the foot surface, both normal and shear stresses, may not necessarily be co-located or happen at the same time^51^, and can be affected by gait styles^52^. Furthermore, only 38% of plantar ulcers develop at peak pressure sites^3^. Whilst acknowledging that peak stresses may not be the best measurement, peak stresses is a common metric used by other researchers to compare plantar stresses. Pressure time integrals (PTI) are generally considered a more comprehensive and robust metric for comparing foot stresses, as it considers both the magnitude of pressure and time duration it is applied, and can be more predictive of ulceration risk than peak plantar pressure alone^53^.

The results of the PTI trends matched the cumulative sum of stress squared trends, which follows expectations as both were a form of summation of stresses. The control participants had a more disproportionate distribution of normal stress PTI, with the calcaneus normal stress PTI being ~ 744 kPa·s higher than at the first metatarsal head which can indicate a more heel strike gait pattern in this group^54^. The participants living with diabetes had a more even distribution of normal stress PTI, with a lower difference of 286 kPa·s between the calcaneus and the first metatarsal head (Table 2), which can indicate a midfoot striking pattern^54^. Our PTI proportions explain the peak stresses, as the participants living with diabetes had a more even distribution of peak stresses between the rearfoot and forefoot locations suggesting a flat midfoot strike, whilst the control participants’ peak stresses showed a more distinct foot rollover “double peak” force pattern, with an initial high peak stresses at the calcaneus, followed by a dip at the metatarsal head and then another high peak stress at the hallux as the foot rolls forward^55^.

We evaluated three hypotheses, all of which led to significant findings that contribute further towards the understanding of the mechanisms of foot biomechanics and DFU development.

No association between peak temperature and peak stress, was found to be true, with weak regression values of R^2^ < 0.15 which is consistent with previous findings of relatively low regression values of R^2^ < 0.26^40^. Yavuz et al. (2015) performed similar analysis leading to conclusions that temperature was not an excellent predictor of peak stress or peak shear. However, we postulated that comparing in this way would not bring significant results as there is no relationship between temperature and peak stress that could be explained through physics. However, in contrast energy exchange is a well-known physics phenomena which could link relationships between heating of tissue and mechanical loading through the first law of thermodynamics. The comparison should be made between the square of stress and temperature; or put another way in terms of mechanical strain energy and thermal energy which show consistent dimensions (Eq. (7)). As such, the comparison should relate to cumulative mechanical energy and cumulative thermal energy, which we sought to evaluate in our second hypothesis.

The change in plantar tissue temperature within an individual is correlated to their plantar cumulative stress squared, was proven to be true, as high correlation values were found (*r*_*s*_ ≥ 0.917) and was statistically significant. Previous experimental in-vitro porcine tissue tests^21^ showed viscoelastic heating with cyclic compressive loading which supports the findings of this study.

This is the first time that correlation relationships have been quantified between plantar tissue mechanical loading from gait and plantar temperature within individuals. This is an important finding in that it provides evidence that plantar tissue thermal regulation is related to gait activity for people living with and without diabetes. However, it does not explain the physiological mechanisms behind it, as outlined in Fig. 1. Impaired thermal regulation could be a mechanism for tissue damage and the formation of diabetic foot ulcers, so we propose further research should focus on this. Secondly, the inter relationship between these parameters provides a mechanism for inference between them. More mature and highly developed lower cost temperature sensing can be utilised to infer normal and shear mechanical stress. Whilst there have been positive recent developments in shear sensing technologies^8,9,11^, it is in its infancy and the authors’ research indicates the importance of calibration^11^.

We conducted a group regression analysis with the change in temperature and cumulative total stress squared and showed higher R^2^ values (R^2^ < 0.36) than when comparing just the peak stress and peak temperature regressions (R^2^ < 0.15). Our approach of comparing energy transfer could be the reason for the stronger correlation. However, this regression finding was not statistically significant, which we propose is due to variation in individuals within the group. A more interesting finding may lie in the disruptions of the regression fit (lower R^2^ values of less than 0.08 or negatively related) in the group living with diabetes, compared to the control group. This again warrants further investigation, with a larger sample to enable statistically significant results from the analysis and with a greater differentiation between groups as many of our participants living with diabetes were very active and able and many of our control group were overweight (which is typical of the general UK population^56^).

People living with diabetes would have a higher change in temperature from gait activity, and slower cooling during rest periods than people without diabetes. This was found to be partially true for the metatarsal head region during gait activity and not true for other locations, and not true for the rest period across any location.

In relation to our third hypothesis, we have shown a novel finding in that the energy comparisons revealed a significant difference in change in temperature at the first metatarsal head between the group of participants living with diabetes and the control group. We found a significantly greater change in temperature increase (35% higher) post gait in participants living with diabetes, despite lower cumulative stresses. (8% lower). This temperature difference was higher (57%) when comparing groups living with diabetes that had more than 50% loss of sensation via the monofilament test at the foot than the control participants with healthy BMI. One explanation for this difference is impaired thermal regulation in people living with diabetes as we know there are physiological changes in blood flow, vessel dilation, vessel calcification, vascularisation and tissue stiffening^57^. The larger difference in comparing the group living with diabetes that had more than 50% loss of sensation at the foot may be further evidence of impaired blood flow and vascularisation affecting thermal regulation, as these participants may have peripheral neuropathy according to monofilament assessment guidelines^58^. There is also evidence that biological tissues are damaged at higher temperatures^26,59^. This would pose a question regarding impaired thermal regulation as a mechanism for the formation of diabetic foot ulceration. A similar indicative but not statistically significant result was also found at the hallux.

Data collected from the calcaneus region of the foot in participants living with diabetes and control group shows a lower cumulative sum of total stress squared and lower increase in temperature in people with diabetes as the principles of thermodynamics would suggest. Interestingly the data shows that when the participants living with diabetes stopped walking the elevated temperature was maintained, whereas in control participants cooling occurred. These results are consistent with the thermal regulation differences at other areas of the foot (metatarsal heads and hallux) between participants living with and without diabetes. This is also consistent with studies on poor heat regulation that have indicated the average blood flow (and thus heat flow transfer) is lowest in people living with diabetes followed by older and young healthy adults^57^.

These temperature differences relate to complex mechanisms described by the pictorial equation in Fig. 1b. Our results clearly show differences at different locations, indicating that gait style, or anatomical changes may be responsible for these relationships. Further work would be required to fully understand the mechanobiological relationships between gait loading, tissue mechanics, heating, vascularisation and neuromuscular biofeedback.

People living with diabetes, especially those with neuropathy, have been found to have altered gait spatiotemporal parameters, such as slower self-selected gait speed, wider step width, or altered proportions of stance and swing phase, all of which could alter gait loading^60,61^. Differences in tissue mechanics have also been reported in people living with diabetes, in that they have increased stiffness and toughness^35^, leading to altered gait biomechanics and joint mobility^62^, and compromised ability to dissipate stresses that may increase ulceration risk in plantar tissues^63^. Vascularisation and neuromuscular feedback are also affected by the diabetic condition. People living with diabetes have differences to healthy individuals in blood vessels, reduced vasodilation and blood flow, which could impact the body’s ability to redistribute blood for temperature regulation^28,64^. These interlinked relationships contribute to the overall heat transfer at the foot, and may present itself as differences in temperature measurements when comparing groups of people living with and without diabetes, and for those with neuropathy.

R^2^ values for the linear regressions were higher in the control group (group C, R^2^ = 0.076–0.361) than the group containing people living with diabetes. (group D, R^2^ = 0.0001–0.081). This indicated that there was a stronger linear relationship between temperature change (heat energy) and cumulative stresses squared (strain energy) in the control group (C). This may also be evidence of irregularity or impairment in temperature regulation in the group living with diabetes.

Finally, as there were no significant differences in walking speed and cadence, there was no indication of these affecting the measurement of temperature or stress. However, these remain important factors to consider as this affects foot loading and thus may affect measurement.

## Study limitations

Study limitations which affect the intra participant analysis is:

(i) We used a Spearman’s rank correlation to determine the relationship, which only assesses whether the relationship is consistently increasing or decreasing. In this study, our strong correlation proved that when strain energy (cumulative sum of stress squared) increases, heat energy (change in temperature) increases as well, but whether it is linear or nonlinear increase is unknown. Future work should consider both linear or non-linear prediction models to further understand this relationship.
(ii) Our study used a lumped parameter model which assumed the plantar tissue has homogeneous properties. This is a limitation as plantar tissue is made from layers of different tissue (skin, fat, muscle, bone, etc.) which will differ in thickness and composition across the regions of the foot. However, our lumped parameter model was sufficient to identify strong correlation between strain energy produced during gait loading and heat energy generated in the plantar tissue. Future studies should focus on the role of different tissues within the plantar tissue and their differences in people living with and without diabetes which may lead to new insights into temperature regulation during gait.

Study limitations which affect the group analyses are:

(i) Our sample size is relatively small and there is not a clear differentiation between the groups of participants living with diabetes and the control participants without diabetes in terms of BMI. Although it should also be considered that the general UK adult population now have high BMI, with a mean of 27.6 kg/m^2^ for both men and women, which is in the overweight category^56^. As higher BMI has links to poor vascularity^65^, the overweight or obese participants in the control group (~ 56% of the participants) may have similar results to the participants in the group living with diabetes, particularly as 25% of these participants had healthy BMIs. Cohen’s D effect size calculation for hallux and calcaneus foot locations for both groups were ~ 0.2, which meant that the participants had high interindividual variability for the sample size of the study. These may have affected the findings in our study where:
  - There was a lack of statistical significance in the comparison of medians of temperature differences at the hallux and calcaneus between the group living with diabetes and control group.
  - Low R^2^ values were obtained in the linear regression analyses between groups’ energy transfer (between cumulative sum of stress squared and temperature change). However, the R^2^ values were higher than when comparing peak temperatures and stresses found in this study, as well as in previous work by Yavuz et al. (2015).
(ii) Moreover, other factors like variation in gait style may have contributed to weaker regressions, with different gait styles leading to different input work and thus affecting the cumulative foot stresses. This may affect heat generation and the measured temperatures.

This potentially presents the opportunity for further work with a larger sample size and differentiated sample populations between the groups, as well as minimising effects of confounding factors (such as gait) by designing experiments that control for these. For example, we advise researchers to measure and consider controlling for differences in vascularisation, which is an important parameter which governs the thermodynamics of the tissue, but was not measured in this study. In depth assessments for peripheral neuropathy and vascular health, such as recording time since diabetes diagnosis and conducting more clinical foot assessments like the vibration perception tests, may aid in the understanding of how the diabetic condition leads to the formation of a foot ulcer. This is necessary to comprehensively explore different phenotypes and potentially obtain more meaningful differences.

## Conclusion

This study has established a statistically significant correlation between plantar tissue mechanical loading and foot temperature within participants during gait. We applied thermodynamic principles to evaluate this relationship, outworked through our novel in-shoe sensing technology (TNS insole) that measures normal pressure, shear pressure and temperature at the hallux, first metatarsal head and calcaneus during gait. Our research has revealed indicative, yet distinct plantar stress and temperature patterns in groups of people living with and without diabetes, even without clinical presentation of foot pathology. Notably, participants living with diabetes exhibited higher first metatarsal head temperatures and impaired cooling across all plantar regions, with those experiencing sensation loss showing greater temperature increases. This may indicate neuropathy-altered thermal regulation in people living with diabetes compared to those without. Our findings emphasise the need for further work to investigate these differences not only between the population groups in this study but for other population groups with varying foot pathology. These findings highlight the complex interplay between gait activity, plantar tissue thermoregulation, and potential diabetic foot ulcer (DFU) risk, laying the foundation for future investigations into plantar tissue biomechanics and more effective DFU risk management strategies.

## Electronic supplementary material

Below is the link to the electronic supplementary material.

Supplementary Material 1

## Data Availability

The datasets generated during and/or analysed during the current study are available in the Mendeley Data repository, and available at DOI: 10.17632/nxfswyw7zz.1.
