# Supplementary material for: In-shoe plantar temperature, normal and shear stress relationships during gait and rest periods for people living with and without diabetes
Source: Sci Rep. 2025 Mar 14;15:8804. doi: 10.1038/s41598-025-91934-9 (PMC11909256; doi:10.1038/s41598-025-91934-9)

# Supplementary Materials

**Supplementary Table 1.** Inclusion and exclusion criteria for recruitment

| Group | Inclusion criteria | Exclusion criteria |
| --- | --- | --- |
| Participants living with diabetes (D) | 1) Above 18 years old  2) confirmed diagnosis of diabetes  3) at risk of developing a DFU  4) capacity to participate and walk for 10 mins without rest  5) Be able to walk for 30 minutes with no pain, no walking aid and taking breaks if  required.  6) no contraindication to walking and physical activity  7) no broken skin on foot  8) do not suffer from a skin condition such as severe eczema or skin allergies 9) Must have sensation on their feet (Rivermead Assessment of Somatosensory Performance, RASP or 10g monofilament test;  10) no Pain affecting ability to walk  11) able to walk without walking aid or orthotic | 1) active or have had previous foot ulceration  2) severe vascular disease  3) lower limb amputation above the level of the ankle  4) in-shoe orthotics consisting of non-compressible materials  5) dementia, uncorrected visual or psychological impairment  6) psychiatric illnesses or social situations limiting compliance with the study  7) inner ear pathology or other serious underlying balance dysfunction;  significant cardiopulmonary or other systemic disease limiting the patient's ability to walk approximately 10 minutes; - body-mass index (BMI) of more than 40 kg/m2  8) Over the age of 75 |
| Control participants without diabetes (C) | 1) Above 18 years old and below 75 years old  2) Able to walk for 30 minutes with no pain, no walking aid, and taking breaks if required.  3) No contraindication to walking and physical activity  4) No broken skin on foot  5) Do not suffer from a skin condition such as severe eczema or skin allergies | There are no exclusion criteria for healthy participants |

**Authors note:** The inclusion criteria asked for at least 10 minutes of walking without rest and able to walk for 30 minutes without pain. Participants were instructed to stop walking and rest whenever necessary and that we aimed to walk for just 15 minutes (half the inclusion criteria). However, all participants were able to walk for the 15-minute test period comfortably without a rest break at their self-selected walking speed.

**Supplementary Table 2**. Definition of variables quantified for the TNS sensor measurement comparison

| Terminology | Definition | Units |
| --- | --- | --- |
| Average peak stress | Median-average of peak stresses of five minutes in the middle of the walking period | kPa |
| Cumulative sum of stress squared | Is the sum of stress squared at each time interval (sampling time) over the entire 15-minute walking period | MPa^2^ |
| Change in temperature | Difference in temperatures measured from the start to end of an activity period (walking or rest) | °C |
| Peak temperature | Maximum temperature at the end of walking | °C |
| Pressure Time Integral | Stress accumulated over time (area under the stress-time curve) | kPa·s |

**Supplementary Table 3.** Treadmill speed and cadence for all participants

| **Group** | **Participant** | Treadmill Speed (ms^-1^) | Left Foot Cadence (Steps/min) | Right Foot Cadence (Steps/min) |
| --- | --- | --- | --- | --- |
| Control group without diabetes (C) | 1 | 0.80 | 85.8 | 85.0 |
|  | 2 | 0.67 | 81.2 | 78.6 |
|  | 3 | 1.04 | 101.3 | 104.0 |
|  | 4 | 1.20 | 100.1 | 104.4 |
|  | 6 | 1.30 | 112.6 | 111.1 |
|  | 7 | 0.32 | 76.6 | 73.7 |
|  | 8 | 0.64 | 87.3 | 92.4 |
|  | 9 | 0.44 | 71.1 | 69.3 |
|  | 10 | 0.6 | 77.4 | 74.5 |
| Participants living with (D) | 1 | 0.95 | 94.7 | 92.4 |
|  | 2 | 0.80 | 108.6 | 109.7 |
|  | 4 | 1.00 | 106.6 | 105.5 |
|  | 5 | 0.46 | 84.0 | 87.7 |
|  | 6 | 0.90 | 85.4 | 83.3 |
|  | 7 | 1.00 | 95.1 | 95.2 |
|  | 8 | 0.80 | 99.4 | 99.5 |
|  | 9 | 0.73 | 105.3 | 106.5 |

**Supplementary Table 4.** Linear regression analyses between peak stresses and peak temperatures over the 15-minute treadmill walk of the two groups containing participants living with diabetes (D) and the control participants without diabetes(C), respectively.

| Participant Group | X variable | Y variable | Location | R^2^ | Y-intercept (°C) | Slope (Pa/°C) |
| --- | --- | --- | --- | --- | --- | --- |
| C (n = 18) | Peak normal stress | Peak temperature | First metatarsal head | 0.1080 | 30.4 | -11.0 |
| D (n = 16) |  |  |  | 0.0130 | 29.8 | 4.4 |
| C (n = 18) |  |  | Hallux | 0.0110 | 29.8 | 2.1 |
| D (n = 16) |  |  |  | 0.0360 | 29.5 | 7.9 |
| C (n = 18) |  |  | Calcaneus | 0.0003 | 29.8 | 0.5 |
| D (n = 16) |  |  |  | 0.0450 | 29.1 | 6.1 |
| C (n = 18) | Peak anterior shear stress | Peak temperature | First metatarsal head | 0.0010 | 28.5 | -2.9 |
| D (n = 16) |  |  |  | 0.0002 | 30.5 | 0.7 |
| C (n = 18) |  |  | Hallux | 0.1280 | 30.0 | 1.5 |
| D (n = 16) |  |  |  | 0.0950 | 32.0 | -14.3 |
| C (n = 18) |  |  | Calcaneus | 0.0602 | 29.4 | 3.5 |
| D (n = 16) |  |  |  | 0.0468 | 30.0 | 9.3 |
| C (n = 18) | Peak posterior shear stress | Peak temperature | First metatarsal head | 0.0003 | 28.4 | -1.7 |
| D (n = 16) |  |  |  | 0.0010 | 30.6 | -1.1 |
| C (n = 18) |  |  | Hallux | 0.0072 | 30.0 | 1.5 |
| D (n = 16) |  |  |  | 0.0960 | 31.9 | -12.8 |
| C (n = 18) |  |  | Calcaneus | 0.1495 | 29.2 | 6.5 |
| D (n = 16) |  |  |  | 0.0120 | 30.1 | 9.7 |
| C (n = 18) | Peak medial shear stress | Peak temperature | First metatarsal head | 0.0030 | 28.6 | -9.4 |
| D (n = 16) |  |  |  | 0.0460 | 31.2 | -26.7 |
| C (n = 18) |  |  | Hallux | 0.0036 | 30.3 | -3.7 |
| D (n = 16) |  |  |  | 0.0450 | 32.1 | -40.7 |
| C (n = 18) |  |  | Calcaneus | 0.1240 | 29.5 | 5.8 |
| D (n = 16) |  |  |  | 0.0001 | 30.6 | 0.5 |
| C (n = 18) | Peak lateral shear stress | Peak temperature | First metatarsal head | 0.0120 | 28.0 | 9.3 |
| D (n = 16) |  |  |  | 0.0340 | 31.0 | -16.0 |
| C (n = 18) |  |  | Hallux | 0.0778 | 29.7 | 13.2 |
| D (n = 16) |  |  |  | 0.0960 | 32.8 | -62.2 |
| C (n = 18) |  |  | Calcaneus | 0.0500 | 30.2 | -14.2 |
| D (n = 16) |  |  |  | 0.0003 | 30.7 | -1.3 |

**Supplementary Table 5**. Group results of linear regression between total cumulative stress squared (strain energy) and change in temperature from start to end of walking (heat energy) of the 15-minute walk. P-values were greater than 0.05

| Group | X variable | Y variable | Location | R^2^ | Y-intercept (°C) | Slope (kPa^2^/°C) |
| --- | --- | --- | --- | --- | --- | --- |
| Control (n = 18) | Total Cumulative Stress Squared | Final Change in Temperature | First Met. Head | 0.103 | 1.7 | 3.341x10^-10^ |
| Living with diabetes (n = 16) |  |  |  | 0.007 | 2.7 | 1.112 x10^-10^ |
| Control (n = 18) |  |  | Hallux | 0.076 | 2.0 | 3.428 x10^-10^ |
| Living with diabetes (n = 16) |  |  |  | 0.081 | 2.7 | -3.775 x10^-10^ |
| Control (n = 18) |  |  | Calcaneus | 0.361 | 1.8 | 9.443 x10^-10^ |
| Living with diabetes (n = 16) |  |  |  | 0.0001 | 3.6 | 1.586 x10^-11^ |

**Supplementary Figure 1.** Median and interquartile ranges of the change in temperatures for group living with diabetes (D) and control group without diabetes (C) during the 15-minute treadmill walk and the 20-minute rest that followed. (a) Shows the temperature changes of the groups within the 15-minute walk, where a higher median temperature difference in the group living with diabetes at the first metatarsal head was observed. (b) Shows the resting period, where the increase in temperature of both groups has slowed down significantly, and have completely cooled at the calcaneus of the control group without diabetes.
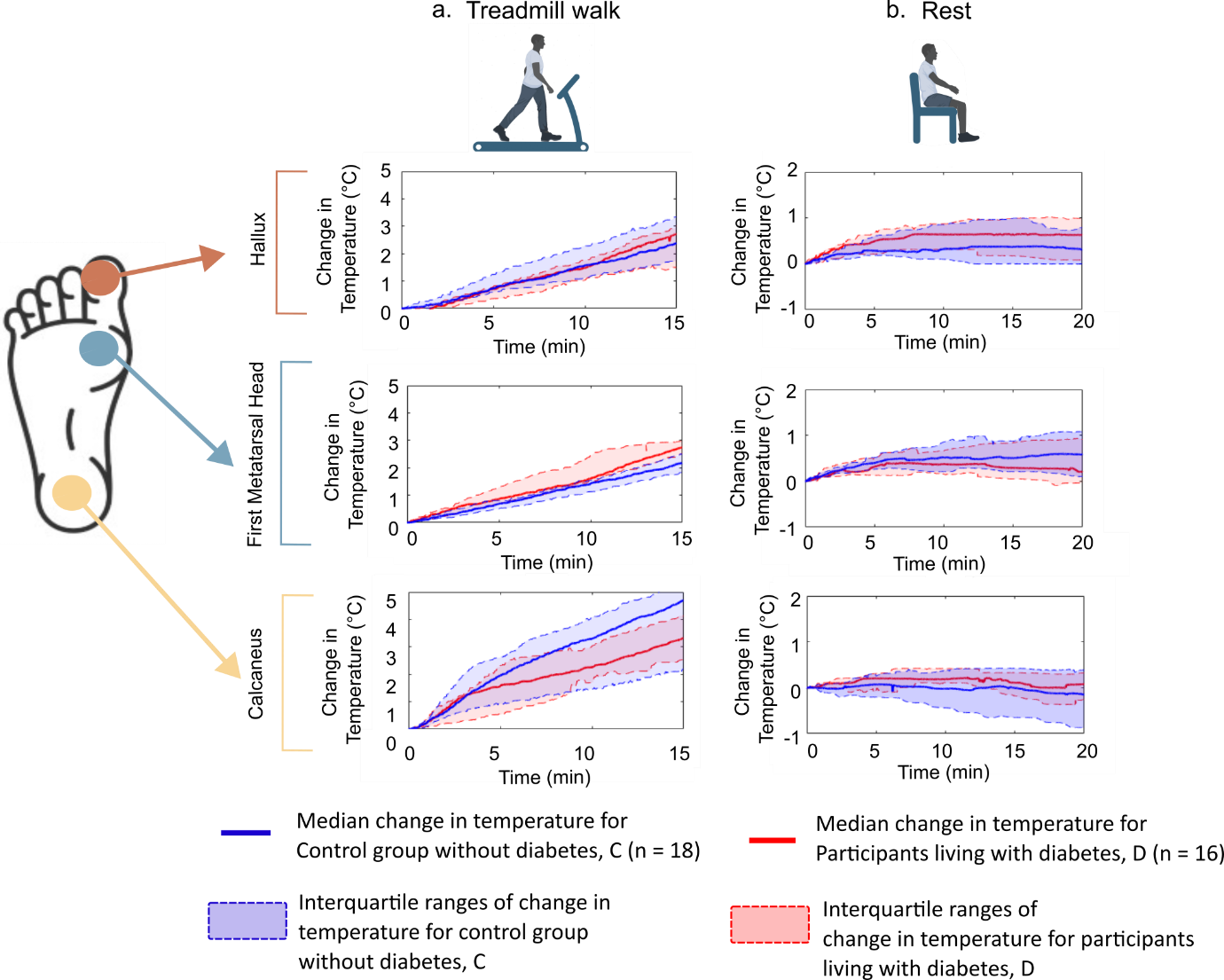


**Supplementary Figure 2**. Intra participant energy analysis. Typical example of a control healthy participant's change in temperature and cumulative sum of stress squared at the sensor locations showing an increasing monotonic relationship, in that the change in temperature increased with the cumulative sum of stress squared.


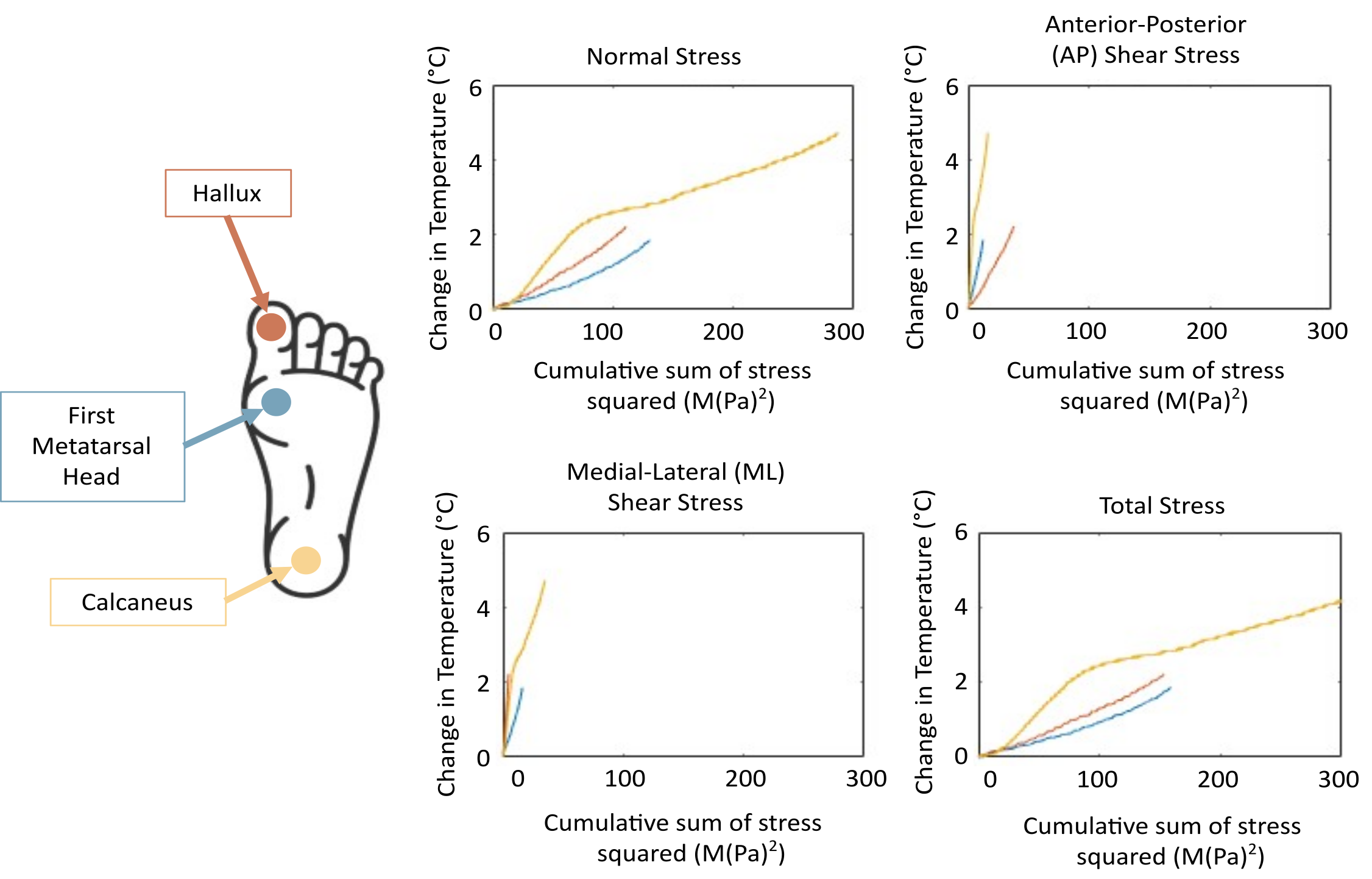

Supplement: Supplementary file 1 — Supplementary Material 1 [file 41598_2025_91934_MOESM1_ESM.docx]
